# Bacterial Cellulose from Tobacco Waste Extract and Its Silver Composite: A Potential Wound Dressing Material

**DOI:** 10.3390/molecules31183270

**Published:** 2026-09-15

**Authors:** Xuewei Niu, Xinlu Yang, Xueru Wang, Jianrong Wu, Minjie Gao

**Affiliations:** Key Laboratory of Carbohydrate Chemistry, School of Biotechnology, Biotechnology of Ministry of Education, Jiangnan University, Wuxi 214122, Chinajmgao@jiangnan.edu.cn (M.G.)

**Keywords:** bacterial cellulose, tobacco waste extract, *Gluconacetobacter xylinus*, wound dressing

## Abstract

The high cost of conventional carbon sources has limited the widespread application of bacterial cellulose (BC). In this study, tobacco stem waste extract (TWE) was evaluated as an alternative substrate for BC production via fermentation with *Gluconacetobacter xylinus*, and a silver-loaded composite (BC-Ag) was further developed for potential wound dressing applications. Medium optimization with the addition of citric acid and (NH_4_)_2_SO_4_ effectively alleviated salt stress and acid inhibition inherent to TWE, achieving a BC yield of 2.40 ± 0.12 g/L while preserving a native cellulose I crystalline structure and three-dimensional nanofibrous network. The resulting TWE-BC exhibited enhanced mechanical properties (ultimate tensile strength: 0.67 MPa; Young’s modulus: 1.56 MPa) and improved thermal stability (glass transition temperature: 58.5 °C). The BC-Ag composite, fabricated via in situ chemical reduction, contained 8.84 wt% silver and demonstrated potent antibacterial activity against *Staphylococcus aureus* and *Escherichia coli*, along with favorable sustained-release behavior. Cytotoxicity assays confirmed good biocompatibility, with cell viability exceeding 70% at extract concentrations of 12.5–50%. In a rat infected-wound model, the BC-Ag dressing effectively eradicated local infection and significantly accelerated wound healing compared to commercial medical gauze, pure BC, and silver-loaded gauze controls. Collectively, this work presents a sustainable and potentially cost-effective strategy for valorizing tobacco waste into a high-value BC-based wound dressing material.

## 1. Introduction

Bacterial cellulose (BC) is a primary metabolite synthesized by bacteria such as *Gluconacetobacter xylinus*, and has a unique three-dimensional nanofiber network structure [1,2], high crystallinity (>80%) [3,4], excellent water retention capacity, and favorable biocompatibility [5,6,7,8,9]. BC has emerged as a promising biomaterial in the fields of tissue engineering [10,11], flexible electronics [12], and medical dressings [13,14]. Compared to plant cellulose, BC is free of lignin and hemicellulose, allowing high-purity products to be obtained without the need for complex purification processes [15,16]. However, the primary bottleneck limiting large-scale industrial application of BC is its high production cost, wherein carbon sources (e.g., glucose) and nitrogen sources (e.g., peptone, yeast extract) in traditional synthetic media account for 30–60% of the total cost [17,18,19,20,21,22]. Against the backdrop of the “carbon peaking and carbon neutrality” goals, exploring the use of low-cost agricultural and agro-industrial waste as alternatives to traditional carbon sources has become a research focus in the global biomanufacturing sector [20,23,24].

Tobacco, as a globally significant economic crop, has enormous annual production, yet the tobacco stem waste generated during processing accounts for up to 25–30% [25,26,27,28]. The abundant cellulose, pectin, reducing sugars, soluble proteins, and mineral elements in tobacco stems render them suitable for microbial fermentation [25,26]. Nevertheless, landfilling or burning is still the primary approach to disposing of tobacco stems, which not only leads to huge losses but also poses ecological risks to soil and water bodies because of alkaloid leakage [29]. While multiple teams have reported on using tobacco stems for BC production, the specific components in tobacco stems, especially nicotine and polyphenols, severely restrict microbial cell wall synthesis and metabolic enzyme systems, causing low fermentation yield and inferior product consistency [30,31,32]. How to systematically optimize fermentation processes to overcome inhibition effects remains a central scientific problem for high-value utilization of tobacco waste.

Although previous feasibility studies have utilized TWE for BC production, mitigating its intrinsic inhibitory effects and achieving biomedical applicability remain significant challenges. To address these challenges, TWE was used as the sole carbon source for BC production. Through a combined approach of single-factor experiments and orthogonal experimental design, the BC fermentation medium was optimized. The resulting BC was characterized in terms of its structural and physicochemical properties. Subsequently, a core–shell BC–Ag composite was fabricated via an in situ chemical reduction method. The microstructure, crystalline nature, antibacterial performance, cytotoxicity, and in vivo wound healing efficacy of the composite were systematically evaluated.

## 2. Results

### 2.1. Analysis of Tobacco Waste Extract Composition

The composition analysis of TWE is presented in Table 1. The extract contained 16.78 ± 0.03 g/L of total water-soluble sugars, 15.80 ± 0.04 g/L of total reducing sugars, 19.5 ± 0.20 g/L of starch, 6.40 ± 0.71 g/L of pectin, and 0.57 ± 0.01 g/L of protein. In terms of the proportion in total water-soluble sugars, glucose accounted for 37.45%, fructose 36.46%, sucrose 16.43%, and maltose 4.23%. These results indicate that TWE is a promising substrate for microbial fermentation.

### 2.2. BC Production with TWE

**Figure 1:**
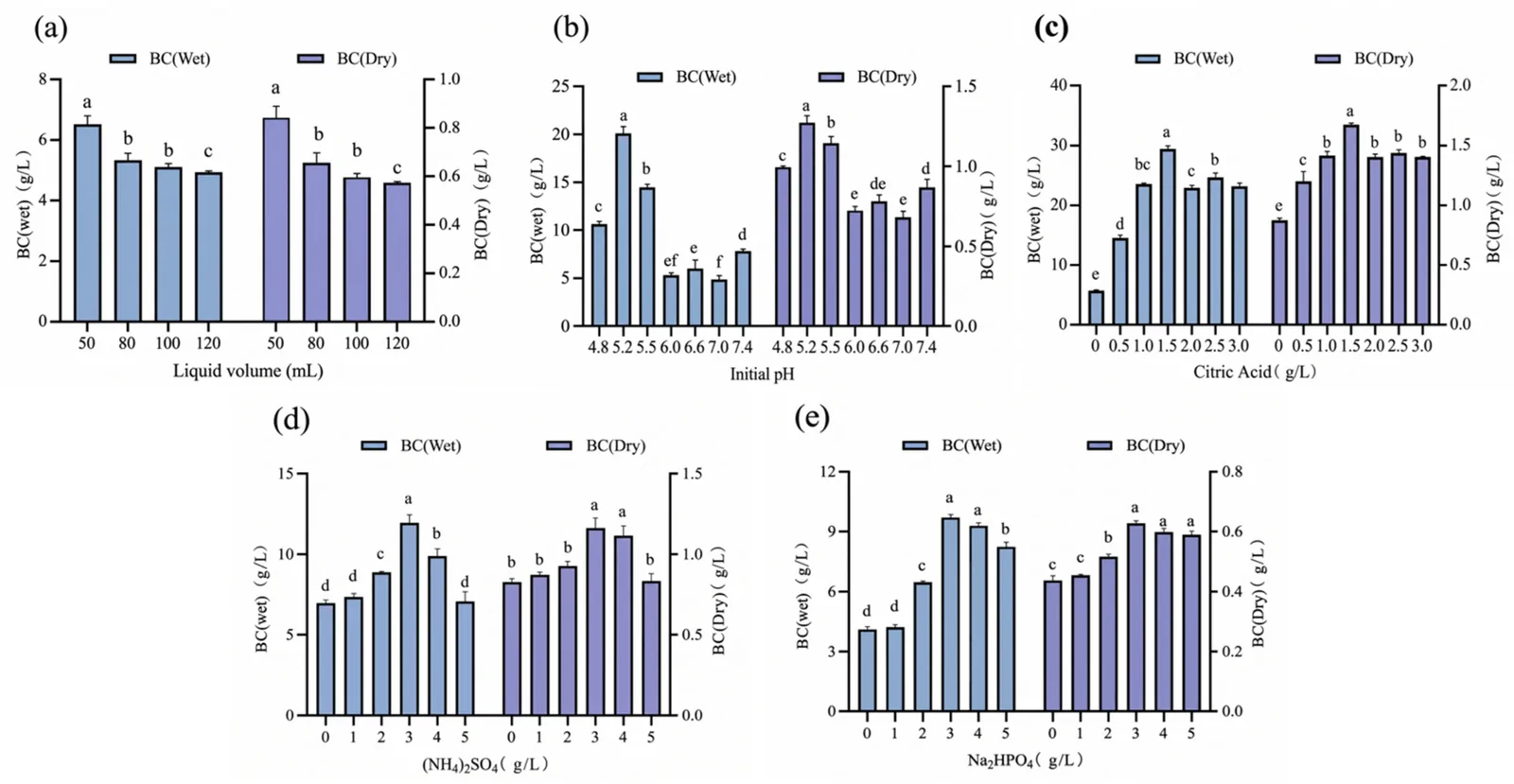
Optimization of BC fermentation with TWE. Note: Data are presented as mean ± SD (*n* = 3). Different lowercase letters (a, b, c, …) above the bars indicate statistically significant differences between groups. (**a**) liquid volume; (**b**) initial pH; (**c**) citric acid; (**d**) (NH_4_)_2_SO_4_; (**e**) Na_2_HPO_4_

Preliminary experiments confirmed that *G. xylinus* can directly utilize tobacco waste extract (TWE) for BC synthesis. To optimize fermentation performance, single-factor experiments were conducted to evaluate the effects of working volume, initial pH, nitrogen source, citric acid, and phosphate source (Figure 1). BC yield decreased monotonically as the liquid volume in 250 mL shake flasks increased from 50 to 120 mL, with the maximum achieved at 50 mL (wet yield: 6.49 g/L; dry yield: 0.83 g/L); hence, this volume was used in all subsequent experiments. Initial pH exerted a strong influence on production: yield rose with pH up to 5.2 (wet: 20.02 g/L; dry: 1.26 g/L) and declined thereafter, with markedly lower dry yields at pH 7.0 (0.68 g/L) and 7.4 (0.88 g/L). (NH_4_)_2_SO_4_ was the most effective inorganic nitrogen source tested and its addition at concentrations of 0–3 g/L steadily increased yield (maximum wet: 11.95 g/L; dry: 1.17 g/L), whereas concentrations above 4 g/L caused a sharp drop. Citric acid supplementation also enhanced BC production, with an optimum at 1.5 g/L (wet: 29.35 g/L; dry: 1.68 g/L), beyond which a slight decrease was observed. Na_2_HPO_4_ addition similarly affected yield, peaking at 3 g/L within the 0–5 g/L range (wet: 9.68 g/L; dry: 0.63 g/L). On the basis of these individual optima, a combined fermentation condition was established: 50 mL working volume, pH 5.2, 1.5 g/L citric acid, 3 g/L (NH_4_)_2_SO_4_, and 3 g/L Na_2_HPO_4_. Fermentation under this integrated formulation produced 1.12 g/L dry BC.

### 2.3. Results of Orthogonal Experimental Design for BC Fermentation (L9(3^4^))

To mitigate antagonistic interactions among the selected variables, citric acid, initial pH, and (NH_4_)_2_SO_4_—identified as the three most influential factors affecting BC yield—were chosen for orthogonal array optimization. Since Na_2_HPO_4_ exerted the least effect on BC yield among the five factors tested and, as an additional electrolyte, would further raise the ionic strength and osmotic pressure of the TWE broth—which already imposes salt stress on *G. xylinus*—its concentration was fixed at a relatively low level of 1.0 g/L. This retained the basic buffering capacity of the medium while minimizing the additional ionic burden, particularly in combination with (NH_4_)_2_SO_4_. An L_9_(3^4^) orthogonal array was then employed to optimize the levels of citric acid, initial pH, and (NH_4_)_2_SO_4_, aiming to establish an improved medium formulation for BC production by *G. xylinus*. Preliminary trials revealed that the combination of high factor levels severely suppressed microbial metabolism. As a result, intact BC pellicles were obtained in only three of the nine experimental runs, with the maximum dry yield reaching merely 1.21 g/L—considerably lower than anticipated. In the subsequent orthogonal round, the (NH_4_)_2_SO_4_ concentration was substantially lowered (0.5–1.5 g/L), the initial pH was appropriately raised (5.0–6.0), and citric acid was evaluated across a range of 0.5–1.5 g/L. The experimental layout and corresponding range analysis results are summarized in Table 2 and Table 3.

The modified orthogonal protocol yielded markedly improved performance. BC biosynthesis proceeded successfully in all nine trials, with dry yields ranging from 1.41 to 2.40 g/L. Range analysis established the following hierarchy of factor influence: A (citric acid, R = 0.603) > B (initial pH, R = 0.346) > C ((NH_4_)_2_SO_4_, R = 0.326). Citric acid emerged as the predominant factor, whereas initial pH and (NH_4_)_2_SO_4_ exhibited comparable effects, both markedly exceeding the error margin associated with the blank column (R = 0.076). The theoretical optimal medium combination was determined to be A_3_B_2_C_1_, corresponding to 1.5 g/L citric acid, an initial pH of 5.5, and 0.5 g/L (NH_4_)_2_SO_4_.

### 2.4. Morphology, Structure, and Mechanical Characterization of BC

#### 2.4.1. SEM Analysis

**Figure 2:**
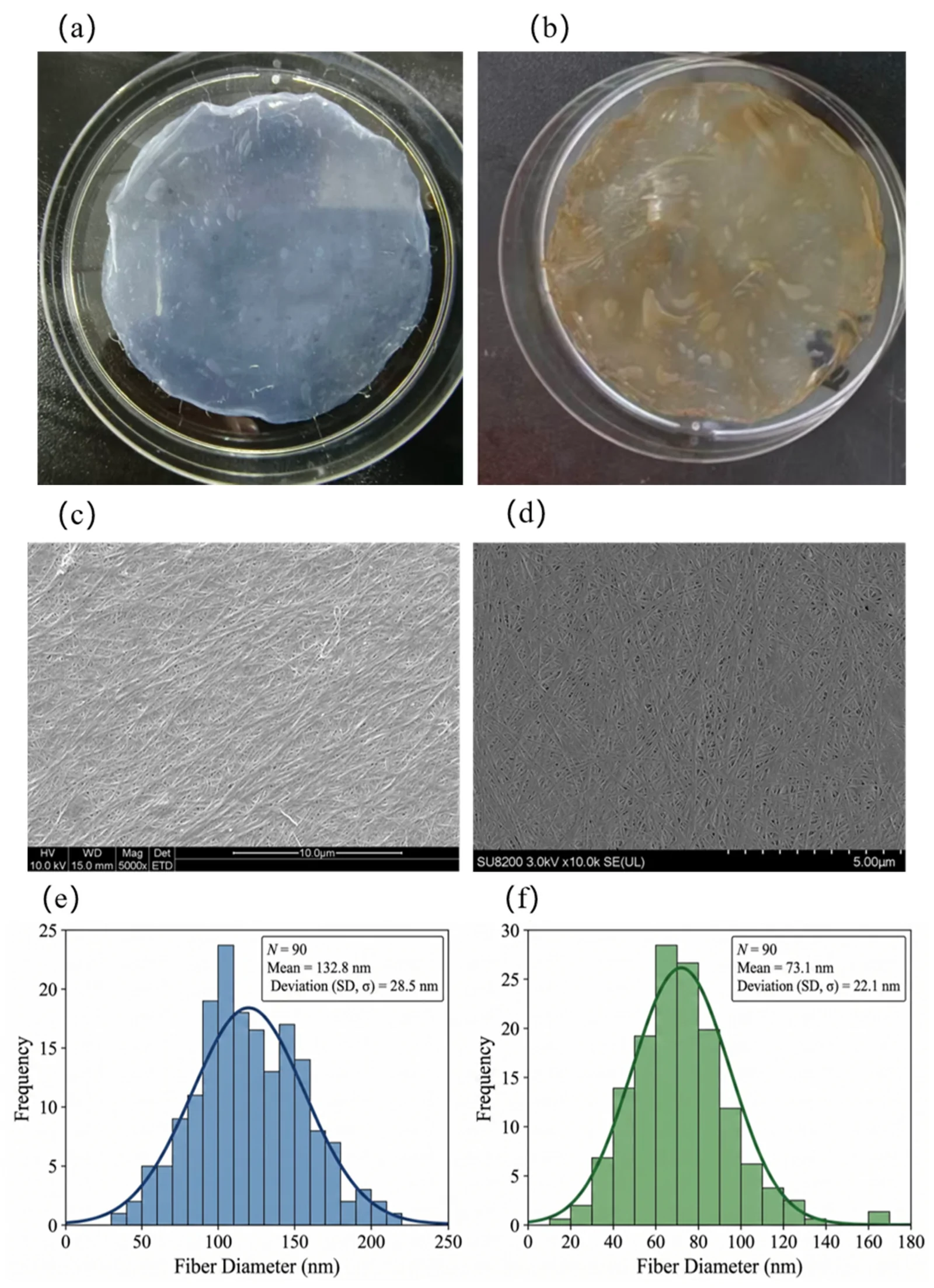
SEM of BC and frequency distribution of apparent diameters. Note: (**a**,**c**,**e**) HS-BC; (**b**,**d**,**f**) TWE-BC.

Figure 2 presents the direct observation and SEM micrographs of BC produced from glucose (HS-BC) and tobacco stem extract (TWE-BC). The HS-BC membrane (Figure 2a) exhibited good transparency, and its SEM images (Figure 2c) revealed uniformly distributed fibers without observable cells or impurities. Likewise, the TWE-BC membrane (Figure 2d) displayed a well-developed fibrous network, free of visible debris or cellular residues. At higher magnification, both samples showed the characteristic three-dimensional porous network architecture formed by numerous intertwined and densely packed nanofibers. Quantitative measurement of 90 randomly selected fibers using image analysis software indicated that both fiber populations followed a normal distribution, but with notable differences in scale. HS-BC fibers were coarser and more broadly dispersed, with an average apparent diameter of 132.8 nm (Figure 2e); approximately 95% of them fell within the range of 75–190 nm. In contrast, TWE-BC fibers were substantially thinner, with a more concentrated diameter distribution centered at a mean of 73.1 nm, and roughly 95% ranged from 29 to 117 nm (Figure 2f). Compared with conventional HS-BC, TWE-BC provided a finer fibrous network and a larger specific surface area, thereby conferring high porosity and a structure favorable for drug loading. The observed morphology indicates that these highly interconnected three-dimensional nanochannels, with their large surface area, confer both outstanding water-retention properties and a suitable structural framework for the subsequent in situ deposition of silver onto BC.

#### 2.4.2. FTIR Analysis

**Figure 3:**
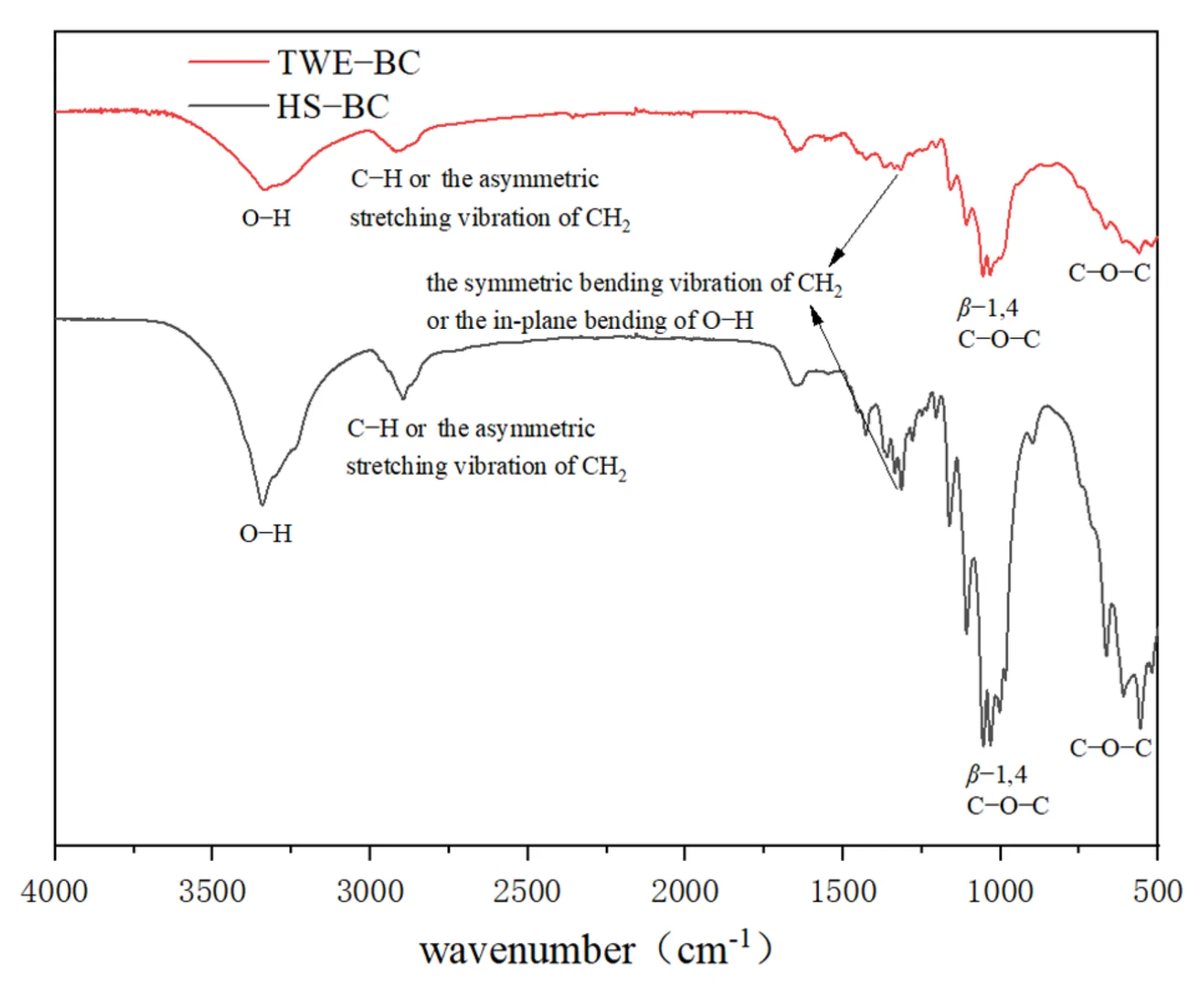
FTIR of HS-BC and TWE-BC.

The FTIR spectra of the BC samples are shown in Figure 3. All BC pellicles displayed the characteristic absorption bands of cellulose. The broad peak around 3300 cm^−1^ was assigned to O–H stretching vibrations, while the band near 2900 cm^−1^ corresponded to C–H stretching vibrations of methyl and methylene groups. The absorption at approximately 1430 cm^−1^ arose from CH_2_ symmetric bending or O–H in-plane bending. A distinct peak at 1160 cm^−1^ was attributed to the antisymmetric stretching of C–O–C in the 1,4-*β*-D-glucosidic linkages. Several overlapping bands in the 1000–1100 cm^−1^ region are associated with C–O stretching of primary alcohols and skeletal vibrations involving C–O–C. Additionally, characteristic BC absorptions between 400 and 1000 cm^−1^ were observed; notably, the signal near 900 cm^−1^ confirmed the presence of *β*-glycosidic bonds in the cellulose structure. Compared to HS-BC, TWE-BC exhibited subtle shifts and intensity variations in specific spectral regions, which reflect the complex fermentation microenvironment of the tobacco extract. The noticeable changes in the O–H stretching region (around 3300 cm^−1^) indicate alterations in the intra- and intermolecular hydrogen-bonding network. This is primarily attributed to the surface adsorption of complex components present in TWE (such as polyphenols and residual proteins), which form new intermolecular hydrogen bonds with the exposed hydroxyl groups of BC. Furthermore, variations in the CH_2_ bending (around 1430 cm^−1^) and C–O–C stretching (around 1160 cm^−1^) regions suggest localized changes in the chain conformation and microenvironment [33]. The steric hindrance caused by these integrated impurities slightly influences the packing of the cellulose chains, an observation that strongly corroborates the minor interplanar spacing increase detected in the XRD analysis.

#### 2.4.3. XRD Analysis

**Figure 4:**
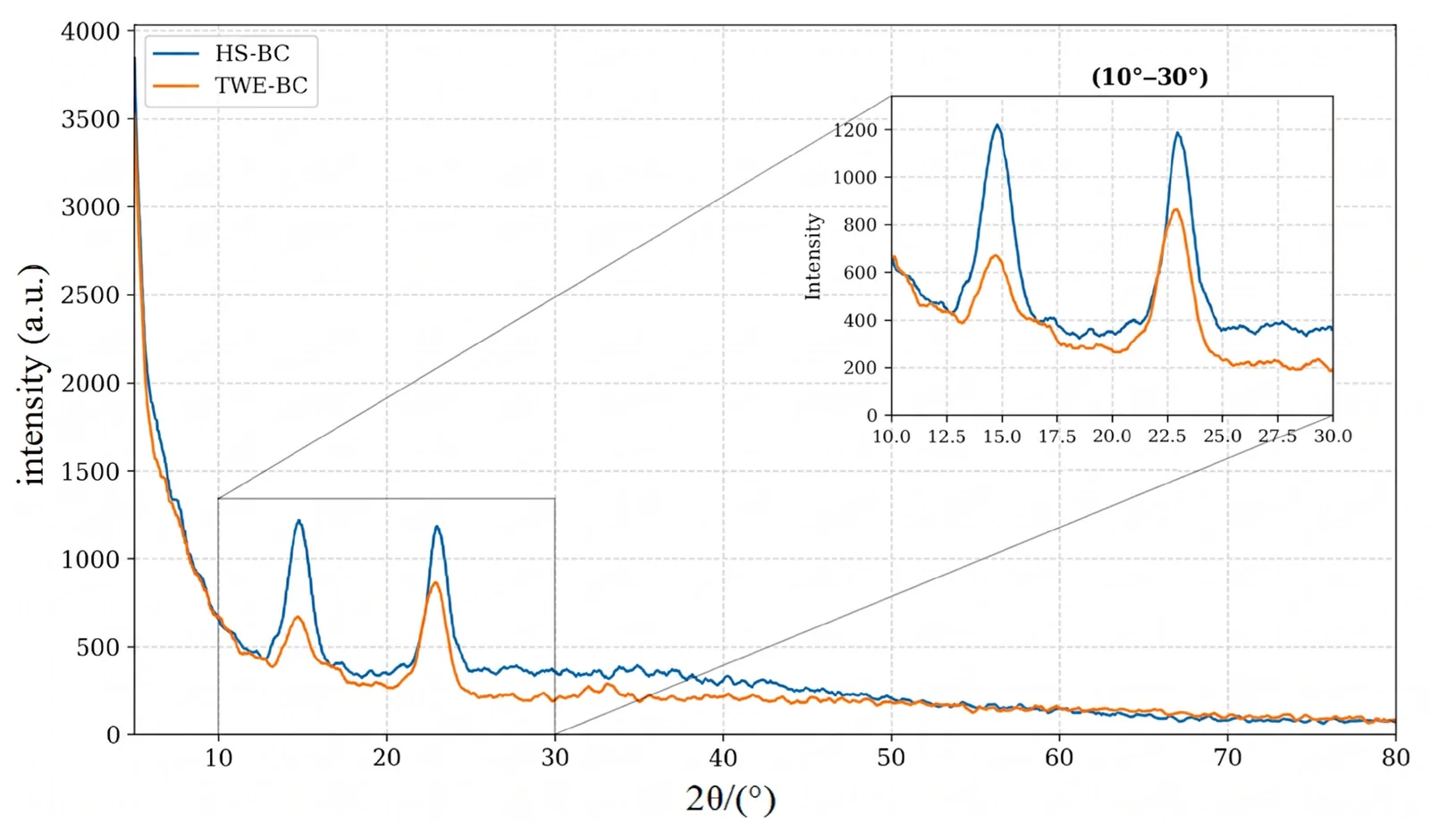
XRD patterns of HS-BC and TWE-BC.

To further investigate the crystalline structure and phase purity of the prepared bacterial cellulose, X-ray diffraction (XRD) analysis was performed over a wide range of 2θ = 5° to 80°. As illustrated in Figure 4 (with the magnified region of 10–30° displayed in the inset), HS-BC has three very typical, high-intensity broad diffraction peaks at 2θ = 14.5°, 16.6°, and 22.97°; TWE-BC also shows highly similar broad diffraction peaks around 2θ = 14.5°, 16.6°, and 22.73°. According to crystallography theory, these three peaks precisely correspond to the characteristic (1$\bar{1}$0), (110), and (200) high-energy diffraction planes of natural Cellulose I.

To further evaluate the crystalline properties, quantitative parameters including the Crystallinity Index (*CrI*) and average crystallite size (*D*) were calculated using the Segal empirical method and the Scherrer equation, as summarized in Table 4. The CrI of the control HS-BC was determined to be 72.83%, with an average crystallite size of 4.34 nm. In contrast, TWE-BC synthesized in the tobacco waste extract medium demonstrated a slightly reduced CrI of 67.74% and a smaller crystallite size of 3.87 nm.

This mild reduction in crystallinity parameters can be attributed to the complex microenvironment of the TWE fermentation broth. During the in situ microbial synthesis, complex non-sugar impurities present in TWE (such as polyphenols, organic acids, and trace residual proteins) tend to adsorb onto the surface or intercalate into the hydroxyl-rich network of the growing cellulose microfibrils. These integrated impurities introduce noticeable steric hindrance, which partially constrains the tight alignment and regular stacking of the glucan chains, thereby suppressing perfect crystal growth. This microstructural perturbation strongly corroborates the subtle shifts observed in the O–H and C-O-C stretching regions during the FTIR analysis. The CrI of TWE-BC remains high (67.74%), maintaining the essential structural integrity, excellent mechanical strength, and fluid-handling capabilities required for high-performance biomedical wound dressings.

#### 2.4.4. Mechanical Property Analysis

**Figure 5:**
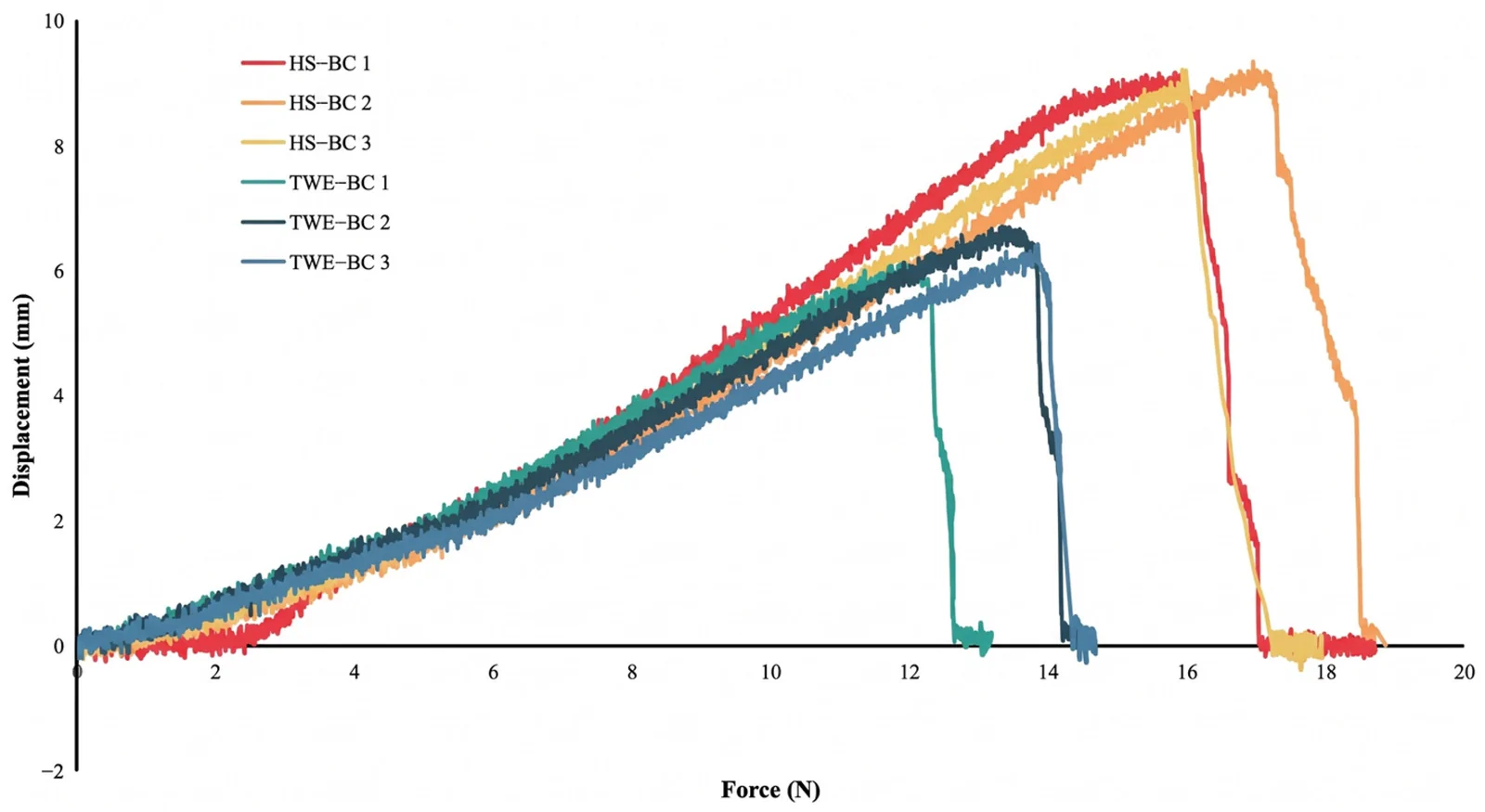
Stretch performance of HS-BC and TWE-BC.

To evaluate the macroscopic mechanical properties of BC produced in different media, tensile tests were performed on HS-BC and TWE-BC (Figure 5), with results summarized in Table 5. TWE-BC exhibited a higher ultimate tensile strength (0.67 MPa) than HS-BC (0.48 MPa). TWE-BC had a significantly smaller cross-sectional area (9.58 mm^2^) than HS-BC (19.25 mm^2^) and thus a lower maximum load (P*_max_*); nevertheless, it attained an ultimate tensile strength (σ) of 0.67 MPa—approximately 40% higher than that of HS-BC (0.48 MPa). Moreover, the Young’s modulus (*E_t_*) of TWE-BC reaches 1.56 MPa, markedly exceeding that of HS-BC (1.04 MPa), indicating enhanced rigidity. This gain in strength and stiffness is accompanied by a moderate reduction in strain at ultimate tensile strength (ε*_m_*), which decreases from 52.70% to 42.23%. Collectively, these mechanical characteristics suggest that TWE-BC possesses a denser, more tightly packed and entangled three-dimensional nanofibrillar network, reinforced by stronger hydrogen-bonding interactions. Such a robust physical scaffold positions TWE-BC as a promising candidate for advanced biomaterial applications.

#### 2.4.5. Thermal Analysis

**Figure 6:**
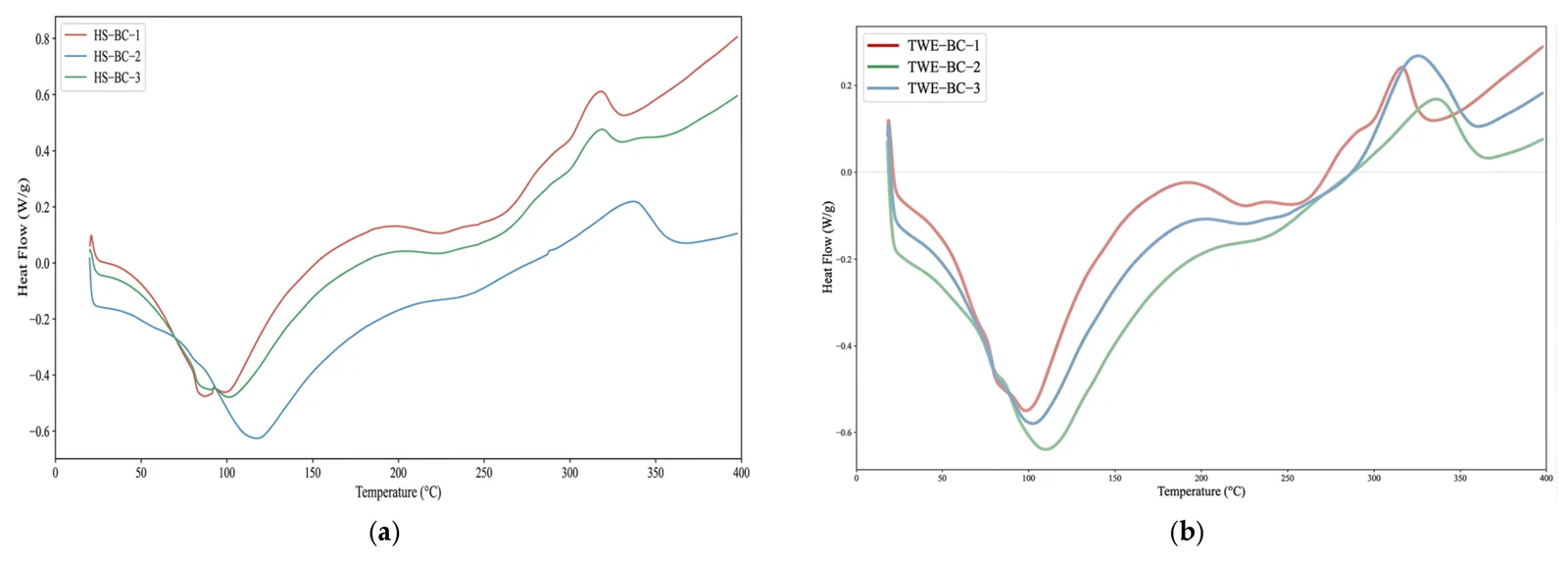
DSC analysis of HS-BC (**a**) and TWE-BC (**b**).

Differential scanning calorimetry (DSC) was employed to assess the thermal stability of HS-BC and TWE-BC (Figure 6). As summarized in Table 6, the glass transition temperature (*T*_g_) of TWE-BC increased notably to approximately 58.5 °C, compared with 48.0 °C for HS-BC. This elevation in Tg may be related to a more densely hydrogen-bonded network, possibly influenced by the complex fermentation microenvironment. Both samples exhibited a characteristic broad endothermic dehydration peak between 87.0 °C and 111.0 °C, indicating comparable water retention capacities within their three-dimensional architectures. Furthermore, the maximum thermal decomposition temperatures (*T*_max_) for both HS-BC and TWE-BC remained within the high-temperature range of 316.0–334.0 °C, corresponding to the cleavage of glycosidic bonds. Collectively, these results demonstrate that replacing the conventional carbon source with TWE does not compromise the intrinsic thermal stability of bacterial cellulose.

### 2.5. Preparation, Characterization and Performance Analysis of BC-Ag Composites

#### 2.5.1. Preparation and Structural Characterization of BC-Ag Composites

**Figure 7:**
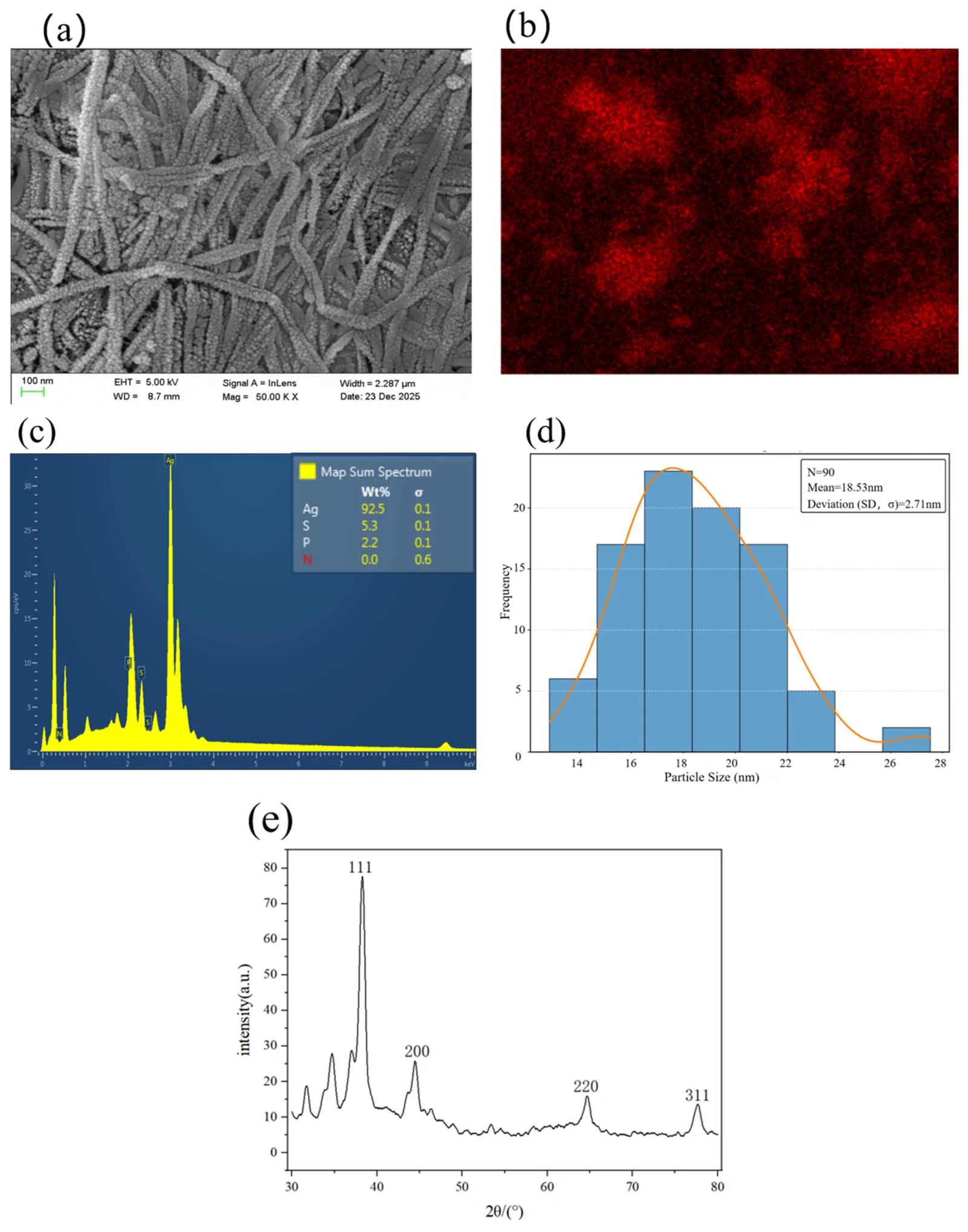
Loading performance of BC-Ag composites. Note: (**a**) surface morphology of BC-Ag; (**b**,**c**) surface silver distribution and content spectrum of BC-Ag (EDS); (**d**) diameter frequency distributions corresponding to (**a**); (**e**) XRD spectrum of BC-Ag.

To further apply BC as a functional biomaterial, BC-Ag composites were synthesized via an in situ chemical reduction method. SEM imaging (Figure 7a) clearly revealed the three-dimensional fibrous network of the composite, with the deposited Ag particles appearing spherical or quasi-spherical. Statistical analysis of the particle size distribution (Figure 7d) yielded a mean diameter of 18.53 ± 2.71 nm. These fine particles formed a uniform and dense sheath-like coating on the BC fiber surfaces—a morphological feature that provides a high specific surface area, which is critical for enhancing antibacterial activity. EDS mapping (Figure 7b,c) indicated a homogeneous distribution of silver across the material surface. As EDS is a semi-quantitative, surface-sensitive technique, the detected surface composition (approximately 92.5 wt% Ag, with minor S and P signals of 5.3 and 2.2 wt%, respectively) should be regarded as indicative of a Ag-rich surface layer rather than as an exact bulk composition.

The XRD pattern of BC-Ag is presented in Figure 7e. In addition to the characteristic peaks of bacterial cellulose, distinct diffraction peaks were observed at 38.1°, 44.3°, 64.4°, and 77.5°, corresponding to the (111), (200), (220), and (311) crystal planes of face-centered cubic (FCC) metallic silver, respectively. A weak diffraction peak near 31.9° may be attributed to silver chloride (AgCl) or residual biological macromolecules.

The total silver content of BC-Ag was further determined by ICP-OES (Table 7). Triplicate measurements gave highly consistent values of 8.82%, 8.87%, and 8.82%, corresponding to an average total silver loading of approximately 8.84 wt%. This bulk value is considerably lower than the surface elemental composition determined by EDS (92.5 wt%). This discrepancy arises from the fundamental differences in the detection principles of the two techniques and, taken together, the two datasets support a core–shell-like architecture of the composite, with a Ag-rich layer deposited on the outer surface of the BC pellicle. EDS is inherently surface-sensitive; under the applied accelerating voltage, the electron beam penetration is limited to a depth of roughly 1–3 μm, making it unsuitable for bulk quantitative analysis. Given that the in situ chemical reduction preferentially deposits silver on the accessible outer surface of the dense BC pellicle, the EDS spectrum predominantly captures this dense Ag layer (the “shell”), resulting in the highly concentrated reading of 92.5 wt%. In contrast, ICP-OES is a bulk analysis method: after complete acid digestion, it measures the total silver mass relative to the entire composite—including the thick, unfunctionalized three-dimensional cellulose core. Consequently, the total silver loading (8.84 wt%) is markedly lower than the surface concentration, reflecting the true overall proportion of silver within the macroscopic dressing.

#### 2.5.2. Evaluation of Antibacterial Properties of BC-Ag Composite

**Figure 8:**
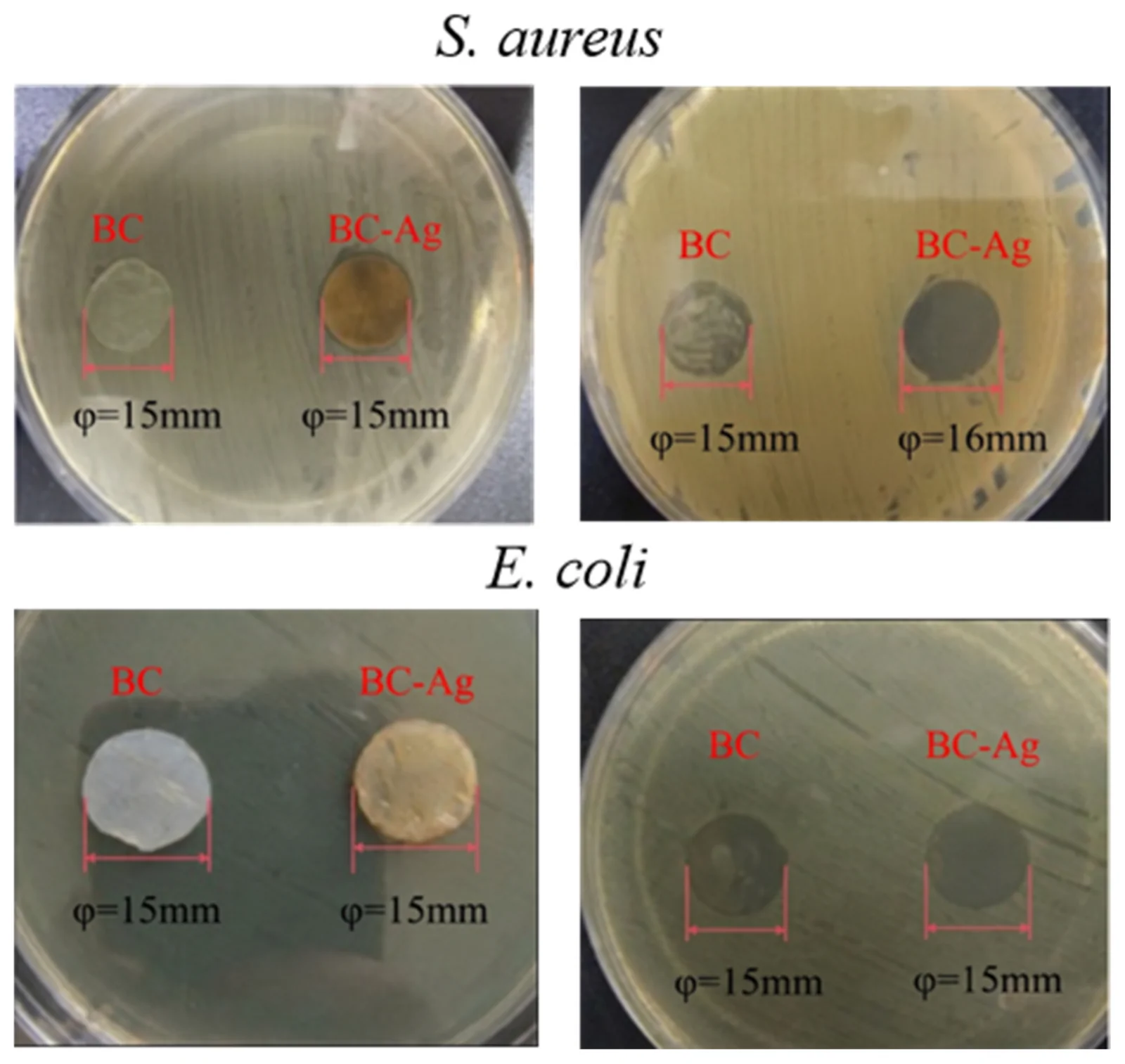
Antibacterial activity of BC-Ag composite against *E. coli* and *S. aureus*.

The antibacterial performance of BC-Ag composites was evaluated against Gram-positive Staphylococcus aureus and Gram-negative Escherichia coli, with pure BC (15 mm in diameter) as the negative control (Figure 8). After 24 h of incubation, the pure BC discs exhibited no inhibitory effect against either strain. In contrast, BC-Ag discs produced a distinct zone of inhibition (φ = 16 mm) against S. aureus, indicating a strong antibacterial effect mediated by the diffusion of released silver species. Against E. coli, however, no extended clear halo was observed; nevertheless, the area directly beneath the BC-Ag disc was completely sterile, confirming potent contact-based growth-inhibitory activity. Collectively, these results suggest that BC-Ag exerts antibacterial action through both diffusion-dependent and surface-contact mechanisms, with the predominant mode varying according to the target pathogen.

#### 2.5.3. Silver Ion Release of BC-Ag Composites

**Figure 9:**
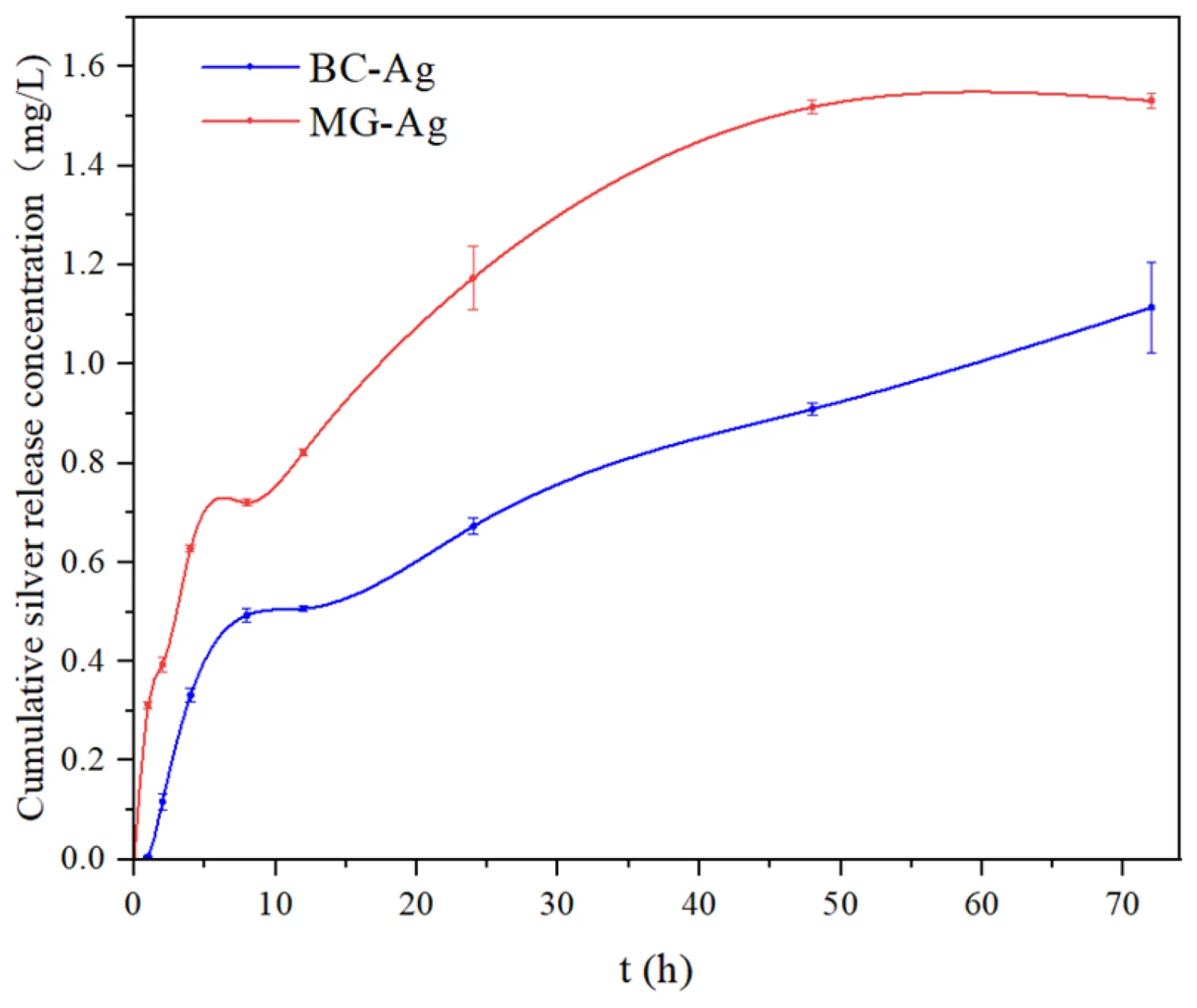
Cumulative silver ion release from BC-Ag composite.

The silver ion release kinetics of BC-Ag composites were investigated, and the results are presented in Figure 9. The medical gauze loaded with silver (MG-Ag) exhibited a pronounced burst release: the amount of released silver increased rapidly within the first 4 h and subsequently remained at an elevated level. In contrast, the BC-Ag composite showed substantially lower cumulative release over the entire 72 h period. A slightly higher release rate was observed during the initial stage compared with later time points, which can be attributed to the relatively weak association of silver nanoparticles deposited on the outermost BC surface. Notably, no obvious burst release was detected for BC-Ag throughout the test duration. The cumulative silver release from BC–Ag reached 1.11 mg/L after 72 h, lower than that from MG–Ag (1.53 mg/L), indicating that BC–Ag composites possess favorable sustained-release characteristics over 72 h, which are desirable for prolonged antibacterial applications.

### 2.6. Cytotoxicity of BC-Ag Composites

**Figure 10:**
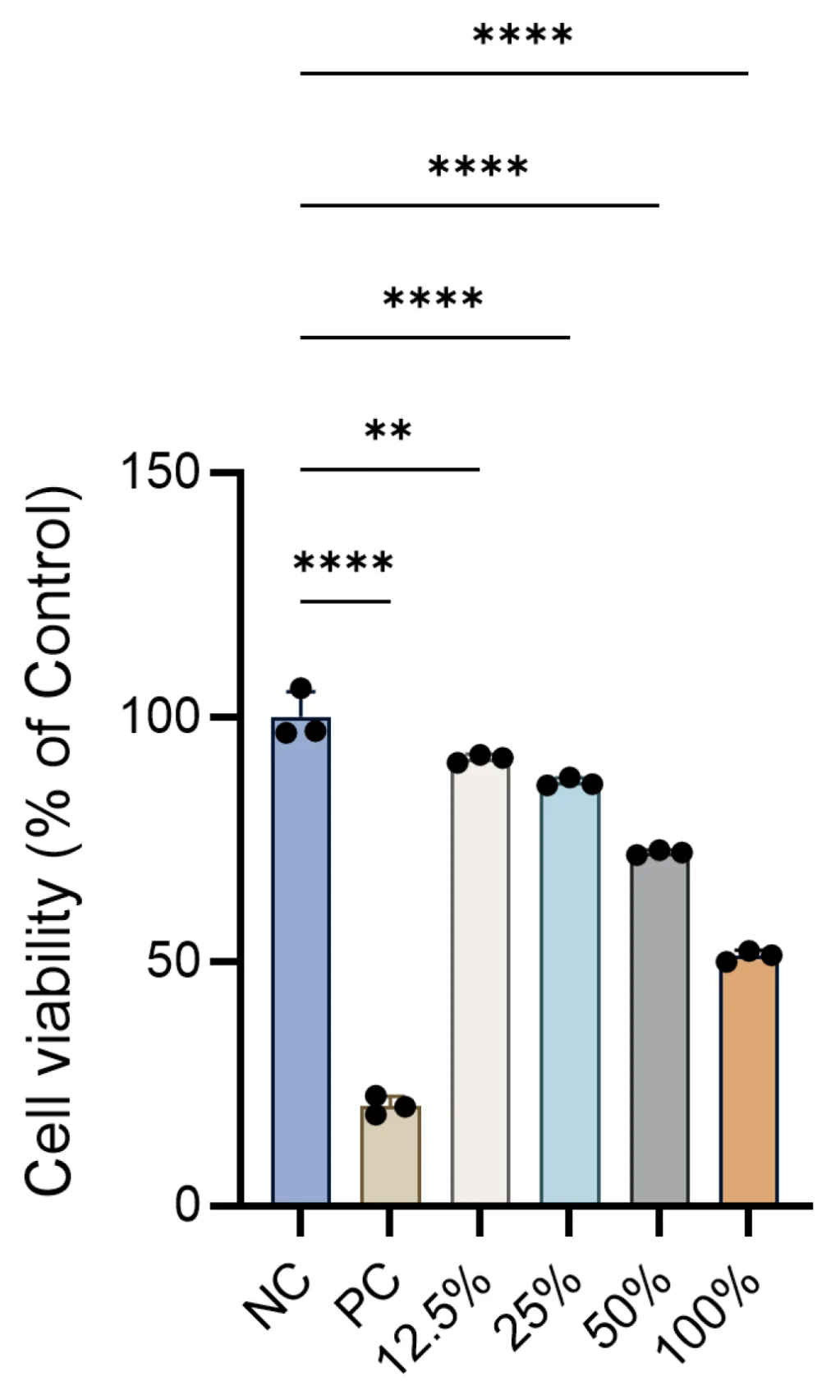
In vitro cytotoxicity of BC-Ag composites. Note: ** *p* < 0.01, **** *p* < 0.0001 vs. NC group.

The cytotoxicity of BC-Ag composites toward L929 murine fibroblasts was evaluated using the CCK-8 assay, with results shown in Figure 10. Assay validity was confirmed by the negative control (NC) group, which exhibited robust cell proliferation (mean OD = 1.060), and the positive control (PC), which showed a marked reduction in cell viability to 20.48% (**** *p* < 0.0001). Treatment with BC-Ag extracts elicited a clear concentration-dependent decrease in cell viability. At extract concentrations of 12.5% and 25%, cell viability remained high at 91.55% and 86.70%, respectively, relative to NC; although the 12.5% group showed a statistically significant difference from NC (** *p* < 0.01), the cell viability was still above 90%. A more pronounced reduction was observed at 50% extract, where cell viability dropped to 72.32%. The highest cytotoxicity was recorded for the undiluted (100%) extract, which reduced cell viability to 51.19% (**** *p* < 0.0001 vs. NC). Overall, these results indicate a concentration-dependent biocompatibility profile, with extracts at 50% or lower maintaining cell viability above the 70% threshold. Importantly, the substantial decrease in viability (51.19%) observed with the undiluted extract highlights the inherent cytotoxicity of high silver concentrations. underscores the importance of the sustained-release core–shell architecture of our BC–Ag composites, which is designed to prevent rapid local accumulation of toxic Ag^+^, thereby mitigating cytotoxicity while preserving antibacterial efficacy.

### 2.7. Evaluation of the Wound Healing Effect of BC-Ag Composites

**Figure 11:**
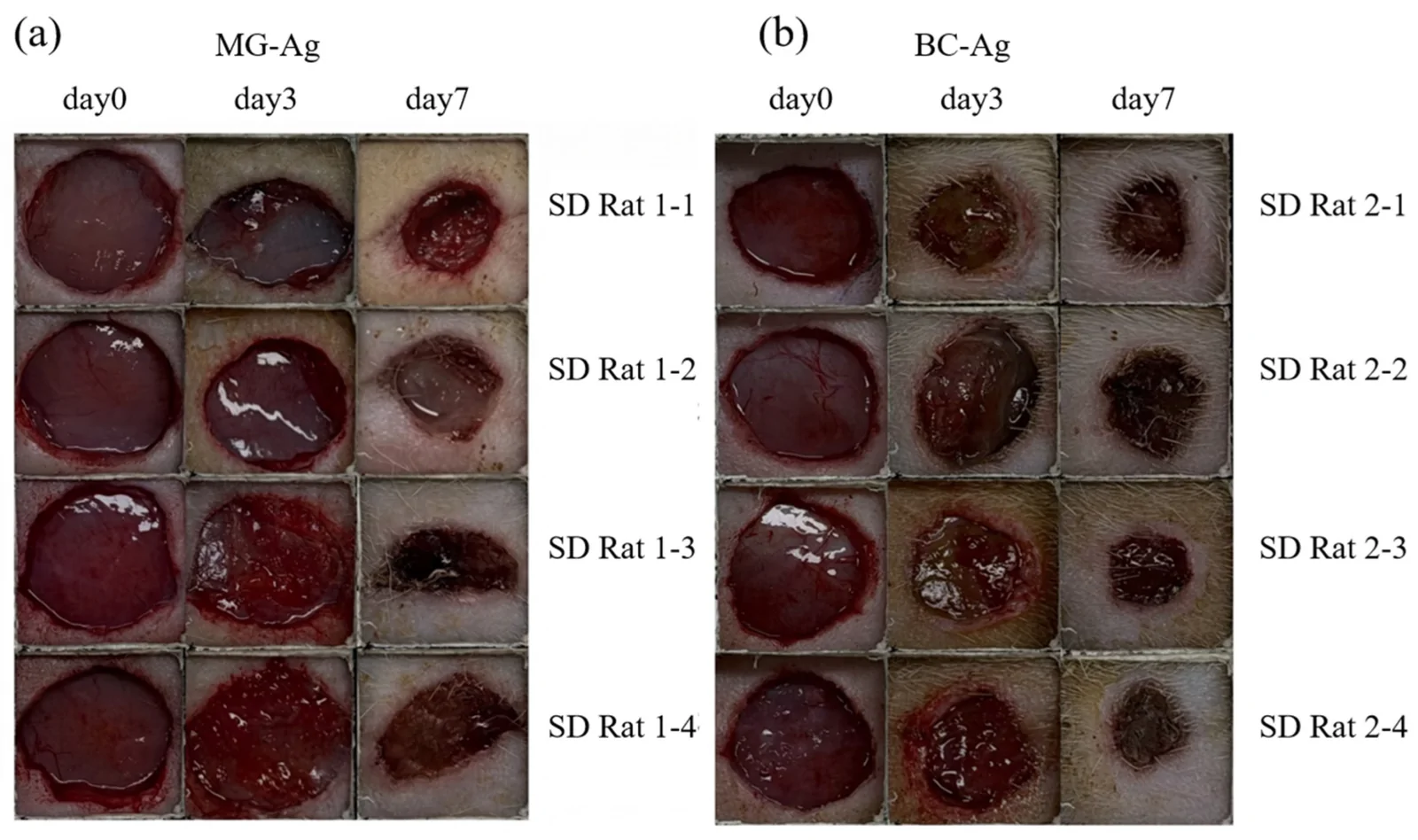

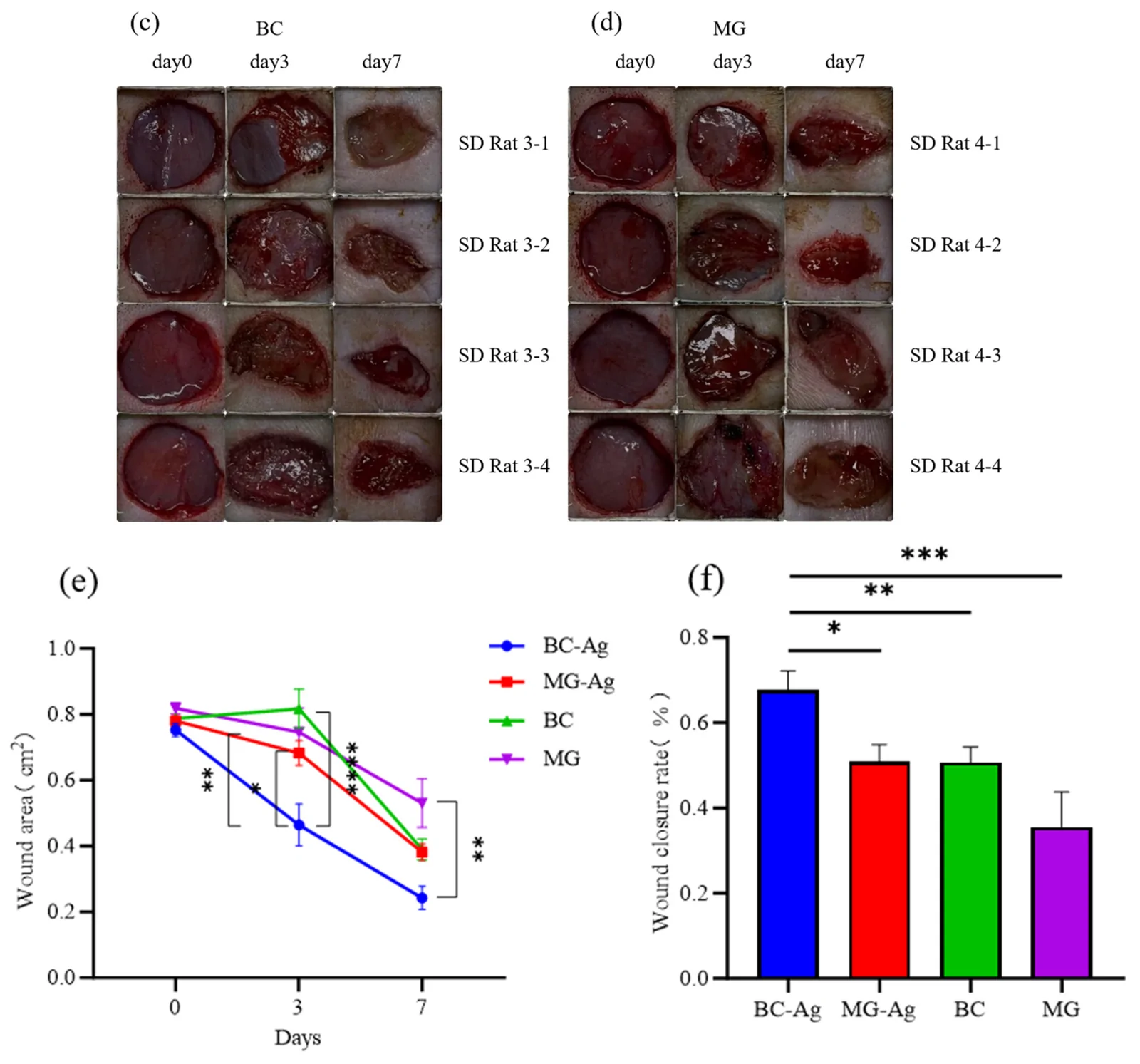
Evaluation of the wound healing capability of BC-Ag composites in a rat model. Note: * *p* < 0.05, ** *p* < 0.01, *** *p* < 0.001, **** *p* < 0.0001 vs. BC-Ag group. (**a**) Wound image treated with MG-Ag; (**b**) Wound image treated with BC-Ag; (**c**) Wound image treated with BC; (**d**) Wound image treated with MG; (**e**) Comparison of wound areas with different dressings; (**f**) Comparison of healing rates with different dressings.

To assess the in vivo wound healing efficacy, a full-thickness skin defect model was established in SD rats (Figure 11a,d). On day 0, all groups exhibited comparable wound sizes with evident exudation. By day 3, distinct healing trajectories became apparent. In the absence of antibacterial agents, both the MG and pure BC groups developed purulent infections, although BC maintained a relatively moist wound bed. The MG-Ag group exhibited pronounced erythema and delayed eschar formation. In contrast, the BC-Ag composite effectively absorbed exudate, formed a uniform scab, alleviated local inflammation, and initiated early centripetal wound contraction. By day 7, the BC-Ag group showed accelerated wound closure with prominent epithelialization and reduced crusting. The pure BC group also demonstrated noticeable late-stage contraction. Conversely, the MG-Ag group retained extensive dark-red crusts, while the blank MG group displayed the poorest outcome, characterized by a large, unhealed wound bed with thick slough and minimal epithelialization, indicating considerably retarded healing.

Quantitative analysis of wound area (Figure 11e) corroborated the visual observations. On day 0, all four groups presented comparable initial wound areas, ranging from 0.75 to 0.82 cm^2^. By day 3, the wound area in the BC-Ag group had markedly decreased to 0.46 cm^2^, which was significantly smaller than those of the MG (** *p* < 0.01) and pure BC (**** *p* < 0.0001) groups, underscoring its early-stage efficacy in wound size reduction. In contrast, the MG-Ag, BC, and MG groups measured 0.68 cm^2^, 0.82 cm^2^, and 0.75 cm^2^, respectively. The pure BC group showed a slight expansion in wound area initially, likely because, in the absence of antibacterial agents, the early acute inflammatory response elicited by *S. aureus* could not be curbed. However, by day 7, the pure BC group demonstrated a strong late-stage healing surge, reducing the area to 0.39 cm^2^ (50.61% closure rate), which was comparable to that of the MG-Ag group (0.38 cm^2^, 50.85% closure rate). The blank MG group displayed the poorest overall healing, retaining a wound area of 0.53 cm^2^ with a closure rate of only 35.54%. Remarkably, the wound area in the BC–Ag group further decreased to 0.24 cm^2^ by day 7 (significantly outperforming the blank MG group in absolute area, ** *p* < 0.01), corresponding to a closure rate of 67.71%. The differences in final closure rates between the BC-Ag group and each of the three control groups (MG-Ag, BC, and MG) were statistically significant (Figure 11f), indicating that BC-Ag not only exerts strong inhibitory effects during the early infection phase but also promotes superior and accelerated healing throughout the entire tissue remodeling process.

## 3. Discussion

Although TWE contains abundant sugars, its complex composition —including alkaloids and polyphenols—creates a highly stressful environment for microbial fermentation. Initial orthogonal experiments for BC production failed, largely because applying a conventional high-nitrogen strategy (suitable for defined synthetic media) led to severe nutrient antagonism in this complex stem-based system [34]. After optimization, the BC yield reached 2.40 ± 0.12 g/L, compared to yields obtained from conventional synthetic media or other agricultural waste substrates reported in the recent literature [32]. By systematically applying single-factor and orthogonal designs, our study revealed that the synergistic addition of citric acid and (NH_4_)_2_SO_4_ effectively alleviated the specific acid inhibition and salt stress inherent to TWE. This targeted optimization strategy successfully overcomes the metabolic bottlenecks identified in previous foundational studies using similar bacterial strains, ensuring robust and reproducible BC production despite the presence of complex non-sugar impurities.

XRD and FTIR analyses confirmed that the non-cellulosic components present in TWE did not alter the BC biosynthesis, as the resulting BC retained the native, highly crystalline Cellulose I structure. The characteristic diffraction peaks of TWE-BC and HS-BC are highly consistent with the standard Cellulose I diffraction patterns widely reported in the previous literature [34]. A minor shift of 0.24° toward lower angles was observed in the (200) diffraction peak of TWE-BC, indicating a slight increase in interplanar spacing. This shift can be attributed to steric hindrance caused by trace amounts of adsorbed hemicellulose or residual proteins on the microfibril surfaces, which mildly disrupted the dense packing of fibrillar bundles without compromising crystalline order [35,36]. The preserved three-dimensional nanofiber network, with its abundant surface-active sites, provides a structurally stable physical scaffold for functionalization and loading applications.

In the analysis of stretching performance, the tensile strength of TWE-BC (0.67 MPa) is comparable to previously reported values for hydrated BC. For instance, hydrated BC typically exhibits tensile strengths ranging from 0.38 to 0.66 MPa [37], with some wet films reporting values up to 1.56 MPa [38]. Our HS-BC baseline (0.48 MPa) aligns well with the lower end of this range [6], while TWE-BC at 0.67 MPa demonstrates a competitive improvement. Regarding Young’s modulus, the water content dramatically affects the mechanical properties of BC. Dry or lyophilized BC can exhibit moduli in the range of 1–1.6 GPa or even up to 130 GPa for single filaments [39,40], whereas our hydrated-state Young’s modulus measurements (1.56 MPa) fall well within the 1–10 MPa range typically reported for fully hydrated BC hydrogels under uniaxial tension [41]. The approximately 50% enhancement in modulus for TWE-BC compared to HS-BC is therefore significant when comparing samples prepared under identical hydration conditions. This moderate decrease in strain at break for TWE-BC (42.23% vs. 52.70% for HS-BC) further supports enhanced structural rigidity; as previously demonstrated, nutrient-induced variations in the culture medium directly alter the BC nanofibril packing density, establishing a clear structural trade-off between elastic modulus and breaking elongation [42]. This observation suggests that using TWE as an alternative carbon source not only maintains but potentially enhances the fundamental mechanical integrity of bacterial cellulose without compromising its characteristic flexibility [6].

The DSC patterns revealed three characteristic thermal transitions for both samples, consistent with previously reported thermal behavior of pure BC. Although Tg values as low as 20–38 °C have been reported for modified or plasticized BC variants, the unmodified TWE-BC exhibited a higher Tg (58.5 °C), reflecting its more densely hydrogen-bonded network [37]. The dehydration event is consistent with the removal of physically bound water within the three-dimensional nano-fibrous network of bacterial cellulose [43]. These decomposition temperatures are significantly above the standard sterilization temperatures required for medical applications (e.g., 121 °C for autoclaving and 180 °C for dry heat sterilization), thereby confirming that both HS-BC and TWE-BC possess sufficient thermal resistance to withstand post-production processing without compromising structural integrity. The reported thermal degradation onset for pure bacterial cellulose typically occurs in the range of 300–375 °C, far exceeding typical autoclave and dry heat sterilization conditions [44,45]. TWE-BC exhibits enhanced thermal rigidity, as evidenced by its higher glass transition temperature (58.5 °C vs. 48.0 °C for HS-BC). The comparable dehydration enthalpies and similarly high decomposition temperatures (316–334 °C) between both samples confirm that the substitution of conventional glucose-based media with TWE does not compromise the fundamental thermal properties of BC. These findings position TWE-BC as a promising sustainable material for downstream biomedical processing, including high-temperature sterilization protocols required for clinical applications.

As for BC-Ag, the significant discrepancy between the surface Ag signal measured by semi-quantitative EDS (92.5 wt%) and the bulk content determined by ICP-OES (8.84 wt%) is consistent with a core–shell-like architecture of the composite, in which silver is preferentially deposited on the accessible outer surface of the BC pellicle. Notably, this bulk silver loading of 8.84 wt% closely aligns with the optimal parameters reported in previous reports [46], which established that a silver content of approximately 10 wt% is ideal for balancing potent antimicrobial action with long-term cytocompatibility. In this structure, BC serves as a three-dimensional skeletal scaffold that ensures mechanical stability, while the silver particles form a dense, active coating on the fiber surfaces. This design is superior to simple bulk blending or doping. Functionally, the dense nanofiber network of BC physically restricts silver ion diffusion, effectively mitigating the burst release behavior commonly observed in loosely packed carriers such as medical gauze. By strategically maintaining the total loading at 8.84 wt%—slightly below the 10 wt% threshold—the composite further minimizes severe silver leaching. This well-controlled release profile avoids the cytotoxicity linked to initial high-concentration bursts, as reflected by the maintained cell viability above 70%, and sustains effective antibacterial silver concentrations over prolonged periods. Regarding the antibacterial performance of BC-Ag, the observed difference in inhibition patterns between *E. coli* and *S. aureus* likely arises from structural differences in their cell envelopes. The dense lipopolysaccharide outer membrane of Gram-negative *E. coli* restricts silver ion penetration, possibly favouring a surface-contact killing mechanism [47]. In contrast, the more permeable cell wall of Gram-positive *S. aureus* is believed to facilitate enhanced ion diffusion, giving rise to a discernible inhibition zone.

The prime criterion for evaluating the safety of antimicrobial medical materials in clinical practice is biocompatibility. According to ISO 10993-5 [48], a material extract is considered non-cytotoxic if the cell viability exceeds 70% of that observed for the negative control. In this study, the cytotoxic effect of BC–Ag was mainly attributed to the release of Ag^+^ ions. Experimental results showed that at extract concentrations of 12.5–50%, cell viability remained above 70%, indicating good cell compatibility within this dose range and acceptable in vitro cytocompatibility under the tested conditions. This suggests a potential concentration window for future application. At 100% extract, cell viability was reduced to 51.19%. This pronounced inhibition is commonly explained by two potential mechanisms: first, silver ions released from the composite may enter cells and trigger the generation of reactive oxygen species (ROS), leading to oxidative stress; second, high concentrations of nanoparticles may directly compromise cell membrane integrity [49]. Although silver exhibits some toxicity at the undiluted concentration, this is often an inevitable trade-off for its strong antibacterial activity in silver-based biomaterials. BC serves as an effective carrier due to its porous network structure, which acts as a slow-release reservoir through both physical adsorption and entrapment, thereby restricting the burst release of silver and ensuring adequate biocompatibility at lower concentrations [50].

In the rat wound healing test, BC-Ag exhibited a significant early healing advantage. The observed day-7 healing rate of 67.71% is highly competitive compared to the recent literature, which typically reports healing rates of approximately 49% to 75% over the same period for conventional BC dressings [51]. Furthermore, this performance significantly outperformed the experimental control groups, including MG-Ag (50.85%), pure BC (50.61%), and blank MG (35.54%). Notably, the pure BC group, despite completely lacking antibacterial agents, performed much better than the blank MG group and achieved a late-stage healing rate comparable to the silver-loaded gauze (MG-Ag) by day 7. This accelerated contraction of granulation tissue and wound closure in the BC–Ag group can likely be attributed to the dual physical and biochemical mechanisms of the composite system. On one hand, the silver-loaded BC is believed to exert broad-spectrum antibacterial effects upon contact with wound exudate, rapidly inhibiting pathogenic bacteria colonizing the wound bed and thereby potentially interrupting the prolonged inflammatory phase driven by continuous bacterial proliferation. On the other hand, the bacterial cellulose substrate, owing to its high porosity and excellent water absorption and retention, is thought to facilitate the drainage of excess inflammatory exudate and to provide a suitable moist scaffold that accelerates macroscopic wound contraction and promotes overall tissue repair via its three-dimensional network. This ultimately helps guide the wound out of the inflammatory phase and into the tissue remodelling stage [52]. This intrinsic moist healing advantage also likely explains the superior late-stage recovery of the pure BC group over conventional dry gauze.

Nevertheless, several limitations of this study should be acknowledged. First, although the disk diffusion assays confirmed contact-based growth inhibition, quantitative time-kill studies and in vivo CFU reduction measurements were not conducted, leaving the bactericidal kinetics incompletely defined. Second, our in vivo assessment was limited to macroscopic wound closure and early-stage (7-day) antibacterial efficacy, without histological or immunohistochemical analyses. Thus, future investigations should incorporate quantitative microbiological assays, extended observation windows (e.g., 14–21 days), and comprehensive histological evaluations (e.g., H&E and Masson’s trichrome staining) to fully elucidate the micro-level mechanisms underlying late-stage tissue remodelling. Notably, while previous work has largely concentrated on the upstream synthesis and fundamental characterisation of TWE-BC, our in vitro and in vivo findings provide new evidence supporting its biomedical potential. Collectively, these preliminary results indicate that this agricultural waste-derived matrix, when functionalised with silver nanoparticles, exhibits adequate biocompatibility and favourable antibacterial activity for early-stage wound management.

## 4. Materials and Methods

### 4.1. Materials

The tobacco stems were provided by Anhui Jiaotianxiang Biotechnology Co., Ltd. (Xuancheng, China). *Gluconacetobacter xylinus* ATCC 23767, *Escherichia coli* ATCC 11775, and *Staphylococcus aureus* ATCC 12600 were all purchased from the Guangdong Microbial Culture Collection Center (GDMCC, Guangzhou, China) and preserved in glycerol stocks at −80 °C. All reagents used were of analytical grade or biochemical grade (Sinopharm, Chemical Reagent Co., Ltd., Shanghai, China).

Hestrin-Schramm medium (HSM, g/L): glucose (20.0), peptone (5.0), yeast extract (5.0), Na_2_HPO_4_ (2.0), citric acid (1.2); pH was adjusted to 6.5.

TWE preparation: The tobacco leaves were removed, and the stems were dried at 60 °C to a constant weight. They were then ground using a grinder. The resulting powder was mixed with deionized water at a 1:10 ratio, extracted at 60 °C for 120 min, and filtered to obtain the extract.

### 4.2. Analytical Methods

The total sugar content in tobacco stems was determined using the phenol-sulfuric acid method, while the reducing sugar content was measured by the DNS method [53]. The total nitrogen content was determined by the Kjeldahl method and converted to protein content using a conversion factor of 6.25. The silver content was analyzed using a UV-Vis spectrophotometer to check the solution’s absorbance, and then analyzed with ICP-OES for silver release from BC-Ag [54,55]. Glucose, fructose, maltose, and sucrose were determined by ion chromatography using an ICS-5000 from Dionex (Sunnyvale, CA, USA). Starch and pectin in TWE were determined according to Yang et al. [56] and Zhang et al. [57].

### 4.3. Experimental Design for BC Fermentation

To optimize the fermentation conditions, single-factor experiments were conducted to evaluate the effects of liquid volume, initial pH, and the addition of citric acid, (NH_4_)_2_SO_4_, and Na_2_HPO_4_ on BC yield.

For liquid volume optimization, 50, 80, 100, and 120 mL of medium were tested in 250 mL conical flasks, with the initial pH fixed at 6.6. After 5 days of cultivation, the BC yield in each group was measured to determine the optimal volume.

To screen for the optimal initial pH, the pH of the medium was adjusted to 4.8, 5.2, 5.5, 6.0, 6.6, 7.0, and 7.4, covering the range suitable for BC production by *G. xylinus*. After 5 days, the BC yield was assessed.

The effect of citric acid was evaluated by adding it at final concentrations of 0, 0.5, 1.0, 1.5, 2.0, 2.5, and 3.0 g/L. The BC yield was determined after 5 days to identify the optimal addition level.

For nitrogen source optimization, (NH_4_)_2_SO_4_ was supplemented at final concentrations of 0, 1, 2, 3, 4, and 5 g/L, and the BC yield was measured after 5 days of fermentation.

To examine the influence of buffer capacity, Na_2_HPO_4_ was added at final concentrations of 0, 1, 2, 3, 4, and 5 g/L, ranging from non-buffered to strongly buffered conditions. After 5 days, the BC yield was measured to determine the optimal concentration.

### 4.4. Orthogonal Experimental Design for BC Fermentation (L9(3^4^))

Based on the single-factor experiments (Section 4.3), citric acid, initial pH and (NH_4_)_2_SO_4_ were selected as the three most influential factors affecting BC yield and were optimized using an L_9_(3^4^) orthogonal array. Na_2_HPO_4_ was fixed at 1.0 g/L owing to its minor effect and to avoid unnecessary ionic strength, and the remaining column of the array was left blank for error estimation. BC yield (g/L) served as the response variable, and each experimental run was performed in triplicate, with the results reported as the mean ± SD of three replicates.

The optimization was performed in two successive rounds. In the first round, three levels of each factor were set, centered on the corresponding single-factor optimum (citric acid: 1.0, 1.5 and 2.0 g/L; initial pH: 5.0, 5.5 and 6.0; (NH_4_)_2_SO_4_: 2.0, 3.0 and 4.0 g/L). In the second round, the factor levels were narrowed based on the outcome of the first round (Section 2.3): citric acid 0.5–1.5 g/L, initial pH 5.0–6.0 and (NH_4_)_2_SO_4_ 0.5–1.5 g/L (Table 2).

Unless otherwise stated, the TWE medium was inoculated with a *G. xylinus* seed culture at 10% (*v*/*v*) and incubated statically at 30 °C for 5 days; these fermentation conditions were applied throughout the optimization experiments in Section 4.3 and Section 4.4.

### 4.5. BC Extraction and Characterization

The BC membrane in broth was harvested, repeatedly rinsed with deionized water and drained. It was then immersed in a 0.2 mol/L NaOH solution and boiled in a water bath at 80 °C for 2 h. Subsequently, the membrane was transferred into deionized water and boiled for another 2 h to completely remove microbial cells and residual medium components. The purified BC membrane was soaked in clean water for 24 h. Part of the sample was freeze-dried for SEM/XRD analysis, while the other part was used in its wet state for mechanical/thermal analysis [32]. The morphology of BC was examined by scanning electron microscopy (Quanta 200, FEI, Hillsboro, OR, USA), and before observation, the freeze-dried samples were mounted on aluminum stubs with conductive adhesive and sputter-coated with a thin layer of gold (approximately 15 nm); observations were carried out at an accelerating voltage of 10 kV. FTIR spectra were acquired using a Nicolet iS50 spectrometer (Thermo Fisher Scientific, Waltham, MA, USA), equipped with an ATR accessory (diamond crystal); spectra were recorded over 4000–400 cm^−1^ at a resolution of 4 cm^−1^, with 64 scans averaged per spectrum, and XRD analysis was performed with a SmartLab diffractometer (Rigaku Corporation, Tokyo, Japan), using Cu Kα radiation (λ = 0.15418 nm) at 40 kV and 40 mA, with a 2θ scan range of 5–80°, a step size of 0.02°, and a scan rate of 5°/min. Furthermore, the mechanical tensile properties of the samples were evaluated using a universal testing machine (LE3504, Lishi Scientific Instruments Co., Ltd., Shanghai, China). Wet BC films were cut into rectangular strips 10 mm in width and 50 mm in length, and mounted with a gauge length of 30 mm. The thickness of each specimen was measured with a digital caliper. The tests were performed at a crosshead speed of 10 mm/min at room temperature; at least three specimens were tested for each sample, and their thermal properties were analyzed via differential scanning calorimetry (DSC 25, TA Instruments, New Castle, DE, USA) using approximately 5 mg of wet sample hermetically sealed in an aluminum pan. The measurement was performed from an initial equilibrium temperature of 20 °C up to 400 °C at a constant heating rate of 10 °C/min. To prevent oxidative degradation during the heating process, a continuous nitrogen (N_2_) purge of 50 mL/min was maintained. The heat flow signals (exothermic plotted upwards) were recorded, and the thermal transition parameters, including glass transition and degradation temperatures, were subsequently analyzed using the TRIOS software (Version 5.4, TA Instruments, New Castle, DE, USA).

### 4.6. Preparation and Characterization of Silver-Loaded BC (BC-Ag)

#### 4.6.1. Preparation of BC-Ag Composites

To test BC as a wound dressing, BC-Ag composite membranes were prepared. First, purified wet BC membranes (15 mm diameter discs) were ultrasonically cleaned in deionized water for 30 min to remove impurities. The BC membranes were then immersed in a 10 mM AgNO_3_ solution and shaken in the dark at 37 °C for 24 h to allow sufficient diffusion of silver ions into the BC pores. After adsorption, the BC membranes were taken out and quickly rinsed with deionized water to remove residual solution. They were subsequently immersed in a 20 mM ascorbic acid solution to reduce Ag^+^ to Ag^0^. The color of the BC membranes changed from white to yellow/brown/dark brown within minutes. The reaction was allowed to proceed for 2–4 h to achieve full reduction. Upon completion of the reaction, the products were washed with water several times until no white precipitate was observed upon addition of sodium chloride solution. The samples were kept wet at 4 °C in a refrigerator or freeze-dried to form dry membranes for subsequent applications [46].

#### 4.6.2. Characterization of BC-Ag Composites

The surface morphology of BC–Ag was observed by SEM (Sigma360, Carl Zeiss, Oberkochen, Germany) at an accelerating voltage of 5 kV after gold sputter coating, and EDS elemental mapping was performed at an accelerating voltage of 20 kV (X-MAX 80, Oxford Instruments, Abingdon, UK).

X-ray diffraction (XRD, SmartLab, Rigaku Corporation, Tokyo, Japan) was employed for phase analysis, and the obtained patterns were compared with the standard reference card (JCPDS No. 04-0783) to identify characteristic peaks. Experimental conditions: Cu-Kα radiation source (λ = 0.15418 nm), tube voltage of 40 kV, and tube current of 40 mA. The detector was operated in continuous scanning mode, with a 2θ scan range set from 10° to 80°, a scan step of 0.02°, and a scan rate of 5° per minute.

To accurately determine the actual silver loading in the composite, acid digestion followed by inductively coupled plasma-optical emission spectrometry (ICP-OES, Agilent 5110, Agilent Technologies, Santa Clara, CA, USA) was performed. (RF Power: 1250 W, plasma gas: 12.0 L/min, auxiliary gas: 1.0 L/min, Nebulizer flow: 0.70 L/min, pump rate: 60 r/min, sample flush time: 20 s, stabilization time: 20 s, reading access time: 5 s. ICP-OES calibration used matrix-matched silver standards (dilute HNO_3_) to eliminate matrix effects. The silver loading was calculated according to the following formulas:

(1) $$\begin{array}{c} C_{x}(mg/kg)\;=\;\frac{C_{0}\left(\mathrm{mg}/L\right)\;\times \;f\;\times \;V_{0}\left(\mathrm{mL}\right)\;\times \;10^{-3}}{m_{0}\left(g\right)\;\times \;10^{-3}}\;=\;\frac{C_{1}\left(\mathrm{mg}/L\right)\;\times \;V_{0}\left(\mathrm{mL}\right)\;\times \;10^{-3}}{m_{0}\left(g\right)\;\times \;10^{-3}} \end{array}$$

(2) $$\begin{array}{c} W(\%)=\frac{C_{x}\left(\mathrm{mg}/\mathrm{kg}\right)}{10^{6}}\times 100\% \end{array}$$

In which:

**M_0_**: Mass of the sample taken for analysis (g)

**V_0_**: Final volume of the sample after digestion and dilution (mL)

**f**: Dilution factor

**C_0_**: Concentration of the element in the test solution (mg/L)

**C_1_**: Concentration of the element in the original sample digest solution (mg/L), calculated as C_1_ (mg/L) = C_0_ × F

**C_X_**: Final test result of the measured element (mg/kg)

**W (%):** Final test result of the measured element

#### 4.6.3. In Vitro Antibacterial Evaluation of BC-Ag Composites

The antibacterial activity of BC–Ag composites was evaluated against *E. coli* and *S. aureus* using a standard disk diffusion assay. Briefly, bacterial strains were cultured in LB broth to the logarithmic growth phase, diluted with sterile 0.9% saline, and adjusted to a working concentration of approximately 1 × 10^6^ CFU/mL. A 100 µL aliquot of the bacterial suspension was evenly spread onto solid LB agar plates. BC discs (15 mm in diameter, negative control) and BC–Ag composite discs (15 mm) were then placed flat onto the agar surface to ensure full contact. After incubation at 37 °C for 24 h, the antibacterial efficacy was assessed by measuring the diameters of the inhibition zones.

#### 4.6.4. Quantification of Total Silver Loading and In Vitro Release

The total silver loading of the solid BC-Ag composites was accurately determined using inductively coupled plasma optical emission spectrometry (ICP-OES) after complete acid digestion of the samples [58]. To simulate wound conditions and assess whether silver release occurs at a rate that could cause cytotoxicity or diminish antibacterial efficacy, a release test was performed. BC–Ag samples were accurately weighed and placed in light-protected centrifuge tubes containing 20 mL of 0.1 M NaNO_3_ solution. This nitrate-based medium was specifically chosen to mimic physiological ionic strength while avoiding the precipitation of insoluble AgCl that would occur in chloride-rich buffers (e.g., PBS or saline), thereby ensuring accurate quantification of released silver ions. The tubes were then incubated in a shaker at 37 °C with agitation at 100 rpm.

At designated time points (1, 2, 4, 8, 12, 24, 48, and 72 h), 2.0 mL aliquots of the release medium were withdrawn for analysis, and an equal volume of fresh, pre-warmed (37 °C) 0.1 M NaNO_3_ solution was immediately added to maintain a constant total volume. Silver concentrations in the collected samples were quantified by UV–Vis spectrophotometry at 490 nm using the rhodanine colorimetric assay. A calibration curve was established prior to the measurements using a series of standard Ag^+^ solutions. To account for the sequential removal and replacement of the medium, the cumulative silver release was calculated by adding the amount of silver detected in the current aliquot to the total mass removed in all previous sampling events. Finally, the cumulative silver release (mg) was plotted as a function of time (h).

### 4.7. In Vitro Cytotoxicity of BC-Ag Against L929 Mouse Fibroblasts

The cytotoxicity of BC–Ag was evaluated against L929 mouse fibroblasts in accordance with ISO 10993-5. Test extracts were prepared following the sample preparation guidelines of ISO 10993-12 [59]. Briefly, sterilized BC–Ag composites (rinsed with deionized water and UV-irradiated on both sides for 60 min) were immersed in serum-supplemented DMEM at a surface area-to-volume ratio of 6 cm^2^/mL and incubated at 37 °C under continuous shaking. The 100% extract was serially diluted with culture medium to 50%, 25% and 12.5% (*v*/*v*). L929 cells in the logarithmic growth phase were seeded into 96-well plates at 1 × 10^4^ cells/well and incubated overnight. After PBS rinsing, the cells were incubated with 100 µL of different extract concentrations. The experimental design included extract groups of BC-Ag, negative control (medium alone), positive control (medium containing phenol), and empty wells as backgrounds. Each experiment was repeated three times. The CCK-8 assay was used to assess cell viability: each well received 10 µL of reagent and was incubated at 37 °C in the dark for 1–4 h. A microplate reader was then used to determine the absorbance at 450 nm [60,61]. The relative growth rate (RGR) was calculated as follows:

(3) $$\begin{array}{c} RGR\left(\%\right)\;=\;\frac{{\mathrm{OD}}_{\mathrm{experimental}}-{\mathrm{OD}}_{\mathrm{blank}}}{{\mathrm{OD}}_{\mathrm{negative}}-{\mathrm{OD}}_{\mathrm{blank}}}\;\times \;100\% \end{array}$$

### 4.8. Wound Healing Capability in a Rat Model

To fully demonstrate the medical application value of BC-Ag composites in real complex physiological environments, a full-thickness skin defect infection model in rats was used to evaluate its wound healing capability [62]. A total of 16 healthy adult male SD rats were selected and acclimatized for one week before surgery. The animals were randomly divided into four groups (*n* = 4 per group): MG (the blank model control group with sterile medical gauze), BC (the model group with pure bacterial cellulose dressing), MG-Ag (the commercial control group with medical gauze loaded with silver nanoparticles), and BC-Ag (the core experimental group with BC-Ag composite dressing). The sample size was chosen in accordance with the 3Rs ethical principles (Replacement, Reduction, Refinement) to minimize animal use at this preliminary proof-of-concept stage, while still permitting statistical evaluation. The rats were then anesthetized, and the dorsal area was prepared by shaving the hair, followed by thorough disinfection of the surgical area with povidone-iodine and 75% alcohol. Under a sterile surgical operation table, using surgical scissors and forceps, full-thickness skin (including the epidermis, dermis, and subcutaneous tissue down to the fascia) was excised symmetrically on both sides of the midline of the rat’s back, creating a circular full-thickness skin defect wound with a diameter of 10 mm. To minimize subjective bias, all subsequent wound photography, area measurements using ImageJ software (Version 1.51j8, National Institutes of Health, Bethesda, MD, USA), and data analyses were performed by an independent investigator blinded to group allocation.

Infection induction: To simulate commonly seen purulent wounds in clinical settings, a logarithmic growth phase *Staphylococcus aureus* suspension (adjusted to a concentration of approximately 1 × 10^7^ CFU/mL) was precisely pipetted, and 20 μL was evenly added to each newly created exposed wound bed. The suspension was allowed to naturally air-dry and penetrate the tissue for 10 min, allowing the pathogenic bacteria to initially colonize the wound bed and induce local infection.

Material application and intervention: Standardized circular dressings with a diameter of 15 mm were tightly applied to fully cover the infected wound and secured on the rats’ torso using sterile 3M medical breathable tape and medical self-adhesive elastic bandages with pressure. The rats were housed individually with access to sufficient sterile water and standard feed.

Macroscopic wound closure rate: On postoperative days 0, 3, and 7, the dressings were removed to expose the wound, and high-definition digital photos of wound healing were taken using a digital camera under fixed height and lighting conditions. At the same time, sterile saline swabs were used to gently wipe away exudate and pus. Image processing software (ImageJ) was used to accurately measure the area enclosed by the edges of the unhealed wound in the photos. The wound closure rate at each time point was calculated according to the formula (%):

(4) $$\begin{array}{c} Wound\;closure\;rate\;\left(\%\right)\;=\;\frac{{\mathrm{Area}}_{\mathrm{Day}\;0}-{\mathrm{Area}}_{\mathrm{Day}\;N}}{{\mathrm{Area}}_{\mathrm{Day}\;0}}\;\times \;100\% \end{array}$$

### 4.9. Statistical Analysis

All experimental data are presented as means ± standard deviations (SD). Statistical analyses were performed using GraphPad Prism 9.0. For the single-factor experiments (Figure 1), differences among treatment groups were assessed by one-way analysis of variance (ANOVA) followed by Tukey’s multiple comparisons test; bars annotated with different lowercase letters (a, b, c, …) differ significantly (*p* < 0.05), whereas bars sharing the same letter are not significantly different. For single-time-point comparisons among multiple groups (e.g., final wound closure rates on day 7), one-way ANOVA was employed, followed by Tukey’s post hoc test. For longitudinal wound area measurements across multiple time points, two-way repeated-measures ANOVA (RM-ANOVA) was applied to account for intra-subject correlation, with appropriate multiple comparisons adjustments. Statistical significance in the figures is indicated as follows: * *p* < 0.05, ** *p* < 0.01, *** *p* < 0.001, and **** *p* < 0.0001 versus the corresponding control group.

## 5. Conclusions

This study demonstrates that tobacco waste extract (TWE) serves as a highly viable and potentially cost-effective substrate for efficient bacterial cellulose (BC) production by *G. xylinus*, achieving a yield of 2.40 ± 0.12 g/L. Systematic physicochemical characterization confirmed that the resulting BC retains the characteristic cellulose I crystalline structure, high crystallinity, and a dense three-dimensional nanofibrous network. Mechanical and thermal analyses further verified its excellent tensile strength and thermal stability. To explore its biomedical potential, BC-Ag composites were successfully fabricated via in situ chemical reduction. The composites exhibited antibacterial activity against both *S. aureus* and *E. coli*, while maintaining acceptable in vitro cytocompatibility under the tested conditions. In a rat full-thickness wound healing model, the functionalized dressing effectively alleviated macroscopic signs of local infection and promoted macroscopic granulation tissue contraction, achieving a wound closure rate of 67.71% by day 7—significantly outperforming the pure BC, silver-loaded gauze, and blank gauze control groups. Collectively, this work presents a cost-effective and sustainable strategy for valorizing tobacco waste into high-value-added antibacterial wound dressings, offering a promising pathway for both waste management and biomedical material development.

## Figures and Tables

**Table 1:** Composition analysis of TWE (1:10, *w*/*v*).

Total Water-Soluble Sugar (g/L)	Total Reducing Sugar (g/L)	Starch (g/L)	Pectin (g/L)	Protein (g/L)	Proportion in Total Water-Soluble Sugar			
					Glucose (%)	Fructose (%)	Sucrose (%)	Maltose (%)
16.78 ± 0.03	15.80 ± 0.04	19.5 ± 0.20	6.40 ± 0.71	0.57 ± 0.01	37.45 ± 0.03	36.46 ± 0.02	16.43 ± 0.02	4.23 ± 0.02

**Table 2:** Factors and levels of the orthogonal experimental design.

Level	A: Citric Acid (g/L)	B: Initial pH	C: (NH_4_)_2_SO_4_ (g/L)
1	0.5	5	0.5
2	1	5.5	1
3	1.5	6	1.5

**Table 3:** Results and analysis of the orthogonal experimental design.

Exp. No.	A: Citric Acid (g/L)	B: Initial pH	C: (NH_4_)_2_SO_4_ (g/L)	D: Blank Column	BC Yield (g/L)
1	1 (0.5)	1 (5.0)	1 (0.5)	1	1.41 ± 0.05
2	1 (0.5)	2 (5.5)	2 (1.0)	2	1.48 ± 0.11
3	1 (0.5)	3 (6.0)	3 (1.5)	3	1.46 ± 0.06
4	2 (1.0)	1 (5.0)	2 (1.0)	3	1.67 ± 0.08
5	2 (1.0)	2 (5.5)	3 (1.5)	1	1.89 ± 0.13
6	2 (1.0)	3 (6.0)	1 (0.5)	2	2.17 ± 0.10
7	3 (1.5)	1 (5.0)	3 (1.5)	2	1.65 ± 0.06
8	3 (1.5)	2 (5.5)	1 (0.5)	3	2.40 ± 0.12
9	3 (1.5)	3 (6.0)	2 (1.0)	1	2.11 ± 0.09
k1 (Mean)	1.45	1.577	1.993	1.803	
k2 (Mean)	1.91	1.923	1.753	1.767	
k3 (Mean)	2.053	1.913	1.667	1.843	
R (Range)	0.603	0.346	0.326	0.076	
Order of influence	1	2	3	-	A > B > C
Optimal level	A3	B2	C1	-	A3B2C1

Note: Data are presented as the mean ± SD (*n* = 3). The k values and ranges (R) were calculated from the mean yields.

**Table 4:** Quantitative XRD parameters of HS-BC and TWE-BC.

Sample	2θ (°)	*I*_200_	*I*_am_	FWHM (°)	Crystallinity Index (*CrI*, %)	Crystallite Size (*D*, nm)
HS-BC	22.97	1185.33	322.1	1.85	72.83	4.34
TWE-BC	22.73	864.35	278.81	2.07	67.74	3.87

**Table 5:** Tensile test of HS-BC and TWE-BC.

Sample	Cross-Sectional Area, (mm^2^)	P*_max_* (N)	Ultimate Tensile Strength, σ (MPa)	Strain at Ultimate Tensile Strength, ε*_m_* (%)	Young’s Modulus, E*_t_* (MPa)
HS-BC	19.25	9.11	0.48	52.70	1.04
TWE-BC	9.58	6.40	0.67	42.23	1.56

Note: The above data are averages and were measured in a wet film state.

**Table 6:** Key thermal properties of HS-BC and TWE-BC.

Sample	T*g* (°C)	T*peak*, Dehydration (°C)	ΔH, Dehydration (J/g)	T*max*, Decomposition (°C)
HS-BC	48	87.6–117.6	207.4–212.9	316.6–334.3
TWE-BC	58.5	99.1–110.8	195.8–212.6	316.3–333.4

**Table 7:** ICP-OES analysis of silver in BC-Ag composites.

Sample Number	Sample Mass m_0_ (g)	Final Volume V_0_ (mL)	Concentration in Test Solution C_0_ (mg/L)	Dilution Factor f	Concentration in Digested Solution C_1_ (mg/L)	Elemental Content in Sample C_x_ (mg/kg)	Elemental Content W (%)
1	0.0020	25	7.06	1	7.06	88,238.05	8.82%
2	0.0020	25	7.10	1	7.10	88,695.41	8.87%
3	0.0020	25	7.06	1	7.06	88,207.78	8.82%

## Data Availability

The data presented in this study are available on request from the author.

## Abbreviations

The following abbreviations are used in this manuscript:

TWE	Tobacco stem waste extract
BC	Bacterial cellulose
SEM	Scanning electron microscope
EDS	Energy dispersive spectrometer
FTIR	Fourier transform infrared spectroscopy
XRD	X-ray diffraction
HSM	Hestrin Schramm medium
TWEM	Tobacco stem waste extract medium
HS-BC	Bacterial cellulose produced on HS medium
TWE-BC	Bacterial cellulose produced on tobacco stem waste extract medium
BC-Ag	Bacterial cellulose loaded with silver nanoparticles
ICP-OES	Inductively coupled plasma-optical emission spectrometer
MG-Ag	Medical gauze loaded with silver nanoparticles
CCK-8	Cell counting kit-8
PBS	Phosphate-buffered saline
DNS	3,5-Dinitrosalicylic acid
DMEM	Dulbecco’s modified eagle medium
